# Isometric Quadriceps Exercises for Patients with Knee Osteoarthritis: A Randomized Controlled Trial Comparing Knee Joint Position Flexion versus Extension

**DOI:** 10.1155/2022/2690871

**Published:** 2022-08-23

**Authors:** Alaattin Sengul, Melek Gunes Yavuzer, Ozal Keles, Ayse Nur Tunali, Deniz Tuncer

**Affiliations:** ^1^Physiotherapy Rehabilitation Department, Institute for Graduate Studies, Halic University, Istanbul, Turkey; ^2^Division of Physiotherapy and Rehabilitation, Faculty of Health Sciences, Halic University, Istanbul, Turkey; ^3^Department of Physiotherapy and Rehabilitation, Institute of Health Sciences, Istanbul Medipol University, Istanbul, Turkey; ^4^Division of Physiotherapy and Rehabilitation, Faculty of Health Sciences, Bezmialem Vakif University, Istanbul, Turkey

## Abstract

**Objective:**

To compare the effect of quadriceps isometric exercises performed in two different positions in addition to the combined physical therapy program on pain, stiffness, and physical function in patients with knee osteoarthritis (OA).

**Methods:**

A total of 30 patients with OA (age range 45 to 70 years) who were admitted to Istanbul Private Ekotom Medical Center, Department of Physical Medicine and Rehabilitation Outpatient Clinic, were included. The patients were randomly divided into two groups according to the type of performing the quadriceps isometric exercises as group 1 (performing in knee extension, *n* = 14) and group 2 (performing in knee flexion, *n* = 15). All patients also received a combined physical therapy program. Exercise protocols were applied six days a week for four weeks. The pain was evaluated using a 10 cm visual analog scale for pain (VAS) in rest and activity; pain, joint stiffness, and physical function were assessed using the Western Ontario and McMaster Universities Osteoarthritis Index (WOMAC).

**Results:**

A significant difference was found in the VAS and WOMAC scores of both groups in group comparisons (*p* < 0.05). When the groups were compared in terms of change values, a significant difference was found in the WOMAC stiffness score in favor of group I (*p* < 0.05). *Discussion*. It is possible to obtain positive results with quadriceps isometric exercises to reduce pain and joint stiffness and increase physical function in patients with knee OA. However, exercises performed in knee extension were found to be more effective in reducing joint stiffness.

## 1. Introduction

Osteoarthritis (OA), which is a chronic degenerative disease characterized by wear on the cartilage structure, causes pathological changes in bone, subchondral bone, and soft tissues and is the most common joint disease that affects patients more than other arthritis [1]. It causes pain, loss of function, and disability affecting the quality of life (QoL) of older adults and represents an increasing health burden on health care resources and society worldwide [2–4].

Although the exact cause of knee OA is not known, the most important risk factors are age, obesity, and knee extensor muscle weakness [5–7]. It occurs in the most load-bearing joints. Therefore, especially the knee joint is the most common OA joint among peripheral joints due to its load-bearing feature [8].

Treatment is based on relieving pain, maintaining or improving mobility, and minimizing disability. Since no drug has the efficacy to change the course of the disease in OA, nondrug approaches and rehabilitation are recommended in the guidelines for the management of knee OA [9–12]. The aim of treatment in OA is to control pain, maintain function with strengthening exercises, protect the joint, keep the damage to a minimum, and increase the quality of life [13]. There are different treatment modalities for chronic diseases (OA, diabetes mellitus, hemodialysis, etc.) used for these purposes in the literature [14–17]. The purpose of an exercise program is to maintain range of motion (ROM), increase muscle strength, and improve overall health. A well-planned exercise program can be as effective as nonsteroidal anti-inflammatory drugs in relieving joint pain [18].

Quadriceps muscle weakness has been suggested to be a risk factor for the development of knee joint OA [19, 20], and previous studies revealed improvements in the relief of joint pain and stiffness and in improving QoL in patients with knee osteoarthritis by strengthening quadriceps muscle [21–23]. Reviewing the literature, it is seen that physical therapy and rehabilitation programs including isometric, isotonic, and isokinetic exercises are applied with different electrotherapy modalities to increase muscle strength for knee OA [24, 25]. The quadriceps muscle is associated with functional tasks such as getting up from a chair, walking independently, climbing stairs, and squatting [7, 26, 27]. It is suggested that quadriceps muscle strengthening maintains beneficial effects in patients with knee OA such as significant reduction of knee pain and improvement of knee function [28, 29]. Of three types of basic therapeutic exercises, isotonic, isokinetic, and isometric exercise, isometric exercise is reported as a simple, safe, and easily performed exercise type which helps accelerate the improvement of strength in muscle [29, 30]. Quadriceps muscle strengthening exercises are reported to be more effective when combined with other electrotherapy modalities [31].

This study hypothesized that quadriceps isometric exercises performed in an extension of knee reduce the severity of pain, joint stiffness, and increase physical function more when performed in flexion of the knee in patients with knee OA. Hence, we aimed to compare the effects of quadriceps isometric exercises performed at two different muscle lengths in addition to the combined physical therapy program on pain, stiffness, and physical function in patients with knee OA.

## 2. Materials and Methods

### 2.1. Study Design

This prospective, comparative study was approved by the Haliç University Non-Invasive Clinical Research Ethics Committee (approval number: 14, 15.03.2012) and conducted following the principles of the Declaration of Helsinki between 2012 and 2013. A verbal and written informed consent was obtained from each participant. A total of 30 patients (7 men, 23 women) (age range 45 to 70 years) who were admitted to the Istanbul Private Ekotom Medical Center, Department of Physical Medicine and Rehabilitation Outpatient Clinic, with knee pain and were diagnosed with knee OA according to the American College of Rheumatology criteria [32] were enrolled and 29 subjects completed the study. Demographic data is given in Table 1.

Inclusion criteria included being of the age between 45 and 70 years, having knee pain for longer than 3 months, absence of socioeconomic and sociocultural barriers to participation in the outpatient treatment program, voluntarily participation in the study, being diagnosed with stage 2-3 unilateral knee OA according to Kellgren-Lawrence scale, and being able to follow the exercise instructions. Exclusion criteria were having a history of previous knee surgery, orthopedic and neurologic problems affecting gait, uncontrolled hypertension, limited range of motion in the hip joint, receiving physiotherapy in the last month, and currently performing strengthening exercises for the quadriceps muscle, contraindication to exercise, and electrotherapy application.

The patients participating in our study were divided into 2 groups of 15, each using a simple random sampling method according to their order of admission. A detailed demographic and clinical information was collected with the “Patient Evaluation Form” prepared by the researchers including age, gender, job, education, body mass index (BMI), and duration of complaints of patients. In the case of bilateral knee OA, the more symptomatic knee was selected for the therapeutic intervention. After the patient was instructed about each test, the following tests were performed before the treatment program and at the end of the fourth week.

### 2.2. Outcome Measures

#### 2.2.1. Visual Analog Scale (VAS)

The visual analog scale (VAS) is a valid and reliable measure of pain intensity [33, 34]. Patients were asked to rate pain intensity by placing a mark on a 10 cm VAS. The VAS was horizontally positioned with the extremes labeled “least possible pain” and “worst possible pain.”

#### 2.2.2. Western Ontario and McMaster Universities Osteoarthritis (WOMAC)

The WOMAC has been widely used by clinicians in the assessment of patients with knee OA since it was developed in 1986 by Bellamy et al. [35] to assess the symptoms of pain, stiffness, and physical function in patients with hip and/or knee OA. It consists of 24 questions and is divided into 3 subgroups: the pain subgroup consists of 5 questions, the stiffness subgroup consists of 2 questions, and the physical function subgroup consists of 17 questions. Each question receives a value between 0 and 4, and the total score is calculated as no difficulty is scored as 0, mild as 1, moderate as 2, severe as 3, and extreme as 4. Higher scores indicate greater physical dysfunction and disability and thereby worse health-related quality of life [36]. The reliability and validity of the Turkish version of the WOMAC questionnaire have also been proven by the study [37].

Our study showed that 14% of the patients were illiterate, which made self-administration of the questionnaire impossible. VAS and WOMAC were administered as reading to be understood by illiterate people.

### 2.3. Treatment Program

Hotpack (HP), ultrasound (US), and transcutaneous electrical nerve stimulation (TENS) were applied to the knees of both groups in a combined physical therapy program for 4 weeks with 6 days a week and 1 session a day, which is a total of 24 sessions. As an exercise program, quadriceps isometric exercises in different positions were performed for both groups, and each patient performed the exercise protocols relevant to their group. The exercises were performed under the supervision of the physiotherapist in the clinic and also were given to all patients as a home exercise program. While the same combined physical therapy program was applied to both groups, quadriceps isometric exercises were applied to the groups in different positions.

#### 2.3.1. The Combined Physical Therapy Program

While the patient was in a sitting position, a combined physical therapy program was applied to one knee of all patients for 4 weeks with 6 days a week and 1 session a day, a total of 24 sessions. Superficial heat application with a hotpack for 30 minutes, conventional TENS (Chattanooga Intellect Legend XT 2 Channel Combination System, USA) for 30 minutes, and therapeutic US (Chattanooga brand) in intermittent mode at a treatment dosage of 3 megahertz, 1 watt/cm^2^ for 5 minutes, were applied to the treated knee. We applied the US in the intermittent mode because it was reported as an appropriate treatment option in patients that have knee OA with cartilage damage [38].

#### 2.3.2. Isometric Exercise Program

Isometric exercises can be performed without need for equipment. Two study groups were defined: group 1 performed isometric quadriceps strengthening exercises in knee extension (quadriceps muscle in short position), and group 2 performed in 90-degree knee flexion (quadriceps muscle in long position). The strengthening exercises were applied as 50 submaximal contractions of 20 seconds and 10 seconds between each contraction. The exercises applied in the clinic were also given to all patients as a home exercise program.

### 2.4. Statistical Analysis

Statistical analysis of the data obtained in this study was performed with the SPSS 15.0 program. Descriptive statistics were used for the demographic and clinical characteristics of the sample, and mean ± standard deviation (mean ± sd) values were given in the tables. Since the distribution was not normal in the sample, a nonparametric Mann–Whitney-*U* test was utilized for comparisons between groups, the Wilcoxon test was used for within-group comparisons, and the chi-square test was used for comparison of demographic ratios between groups. The values obtained before and after the treatment were compared with the Wilcoxon test to show the effectiveness of the applied treatment. In order to understand the superiority of the treatments applied to each other, the difference between the values before and after the treatment was calculated in each group. The groups were compared with the Mann–Whitney-*U* test for this difference. The statistical significance level was set as *p* < 0.05.

## 3. Results

**Figure 1:**
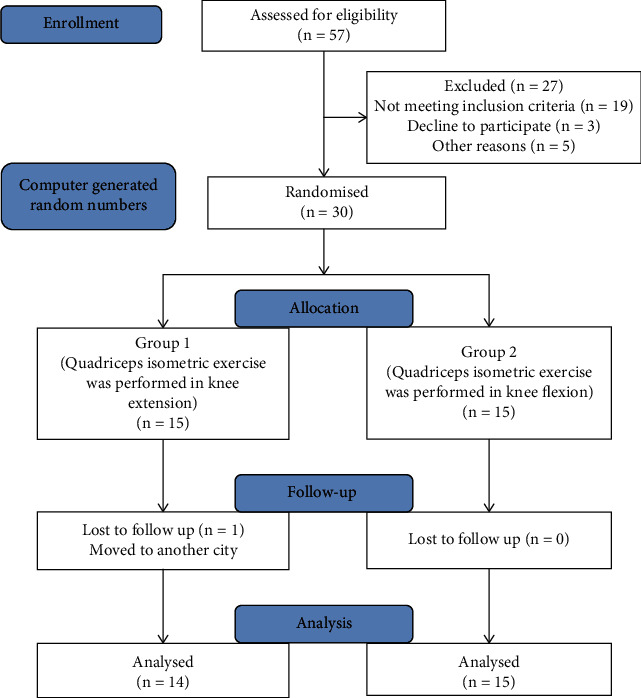
Flow diagram of the study.

The flow diagram of the study is shown in Figure 1. Thirty patients were included in the study. One patient in group 1 could not complete the treatment due to personal reasons. Therefore, statistical analysis was conducted with 29 patients in total.

There were no significant differences in demographic and clinical characteristics of the patients (*p* > 0.05) (Table 1). When the groups were compared in terms of baseline scores of VAS (rest, activity) and WOMAC (pain, stiffness, physical function, and total score) no statistically significant difference was found (*p* > 0.05) (Table 2).

When the scores of VAS (rest, activity) and WOMAC (pain, stiffness, physical function, and total score) were compared within the group at pre- and posttreatment, a statistically significant improvement was found in both groups (*p* < 0.05). Significant improvements in the scores of VAS (rest, activity) and WOMAC (pain, stiffness, physical function, and total score) were observed in both groups after the interventions (*p* < 0.01). In the comparison of the tests between the groups, it was found a significant improvement in the score of WOMAC-stiffness (*p* = 0.035) in favor of group I (Table 3).

## 4. Discussion

In this study, it was observed that isometric exercises performed on the quadriceps muscle of different lengths (knee; 0° extension and 90° flexion) in patients with knee OA were equally effective on pain intensity and physical functions, while improvement in joint stiffness was more remarkable in a knee extension group. To the best of our knowledge, this is the first study that evaluates the effects of quadriceps isometric exercises on two different lengths of the muscle.

Weakness of the quadriceps femoris muscle has been observed in patients with knee OA in several clinical studies [39]. Therefore, strengthening the quadriceps femoris muscle, which is an important stabilizer of the knee, is important in the treatment of knee OA. It has been shown in various studies that an increase in quadriceps muscle strength can be achieved with strengthening exercises and that this will have a positive effect on pain, functional capacity, and gait pattern [40, 41].

Maurer et al. [42] examined the effectiveness of isokinetic and isometric exercise programs in patients with knee OA and reported that both programs achieved significant results on patients. Messier et al. [43] compared the flexion and extension isokinetic strength of 15 patients with OA in the knee joint and 15 healthy individuals, and it was found that there was a statistically significant decrease in the muscle strength of patients with OA compared to healthy individuals. In a controlled study on patients with knee OA conducted by Tan et al. [44], it was stated that there was a decrease in isokinetic and isometric maximal muscle measurements in both flexor and extensor muscles of the knee in individuals with knee OA compared to healthy individuals.

Many studies have measured quadriceps femoris strength in a sitting position, where the muscle is short and weak in strength. However, patients with OA have difficulty in activities that require more muscle length, such as climbing stairs and getting up from a chair [45]. Fisher et al. [46] reported that maximal isometric torque, with regard to muscle length, decreases with the effect of age, and torque increases with increasing hip flexion, and the greatest peak torque is obtained at 0° hip extension angle. However, no difference was found between the peak torque values of 90° flexion and 0° hip extension between the ages of 60 and 70 years.

When the studies that were carried out on different muscle lengths are examined, there are debates about which position the isometric strength of the muscle will be higher. The patients in our study were given isometric strengthening exercises in two different lengths of the quadriceps muscle (knee; 0° extension, 90° flexion). The results obtained from our study show that isometric exercises performed in two different lengths of the quadriceps muscle were equally effective in reducing pain intensity and increasing physical function. It was observed that the exercises performed in the short position of the quadriceps muscle were more beneficial than the exercises performed in the long position of the quadriceps muscle in reducing joint stiffness.

There is currently no definitive treatment for OA. Because pain is the most important cause of disability, treatment options have focused on improving symptoms. However, the factors affecting pain and disability are different from each other. Generally, treatments are more successful in reducing pain than in disability. Exercise has a very important place in knee OA, increasing muscle strength, range of motion, and aerobic capacity, reducing pain, and disability [47].

Özdinçler et al. [48] evaluated the pain level of patients with knee OA with VAS and then included them in a rehabilitation program. At the end of the treatment, they found that there was a significant reduction in pain values. On the other hand, Van Baar et al. [47] divided 201 patients with knee or hip OA to two intervention groups. Both groups received treatment including patient education and medication if necessary. The experimental group also received exercise therapy for 12 weeks. They found that exercise therapy was effective in reducing pain and disability. In our study, the pain level of the patients was evaluated using VAS (rest, activity). After the treatment methods were applied, when the VAS rest and activity scores were compared before and after the treatment, a significant decrease was observed in both groups compared to the initial evaluations. The reason why we obtained positive results in a short time in our study may be a result of the combination of combined physical therapy program and exercise therapy together and the inclusion of outpatients with radiological stages 2 and 3 in the treatment.

Eyigor [49] applied isokinetic and progressive resistive exercises for 6 weeks in patients with knee OA, evaluated the patients with WOMAC before and after the 6-week exercise program, and stated that there was a significant improvement in WOMAC scores at the end of the treatment. Deyle et al. [50] compared 83 patients with knee OA who were randomized as receiving manual therapy and standardized knee exercise program in the clinic and at home and the placebo-controlled group. Both groups were treated twice a week for four weeks. They stated that after treatment, there was a significant improvement in WOMAC pain and daily living activities scores in the exercise group compared to the control group.

At the end of the fourth week of our study, when the WOMAC pain, stiffness, physical function, and total scores were compared before and after treatment, a significant decrease was observed in both groups compared to the first evaluations. When the groups were compared in terms of value changes, a significant improvement was found in the WOMAC joint stiffness score in favor of the group studied in extension (group I). The positional comfort of quadriceps isometric exercises performed while the knee is in extension has increased the effectiveness of the exercise. The reason why the decrease in joint stiffness in the patients in the knee flexion group was less than that in the extension group might have a bearing that the patients had difficulty in learning and performing the exercises. Furthermore, we think that the group working in flexion hindered home exercise programs due to difficulties in doing the exercises.

Gürer et al. [51] investigated the effects of physical therapy methods (HP, US, TENS, and quadriceps isometric exercise) on pain and activities of daily life of patients with knee OA (40 study group; 41 control group). The study group received diclofenac sodium 100 mg/day along with the physical therapy. The same medications without physical therapy were given to control group patients. As a result, it has been determined that the use of physical therapy agents together with nonsteroids and analgesics is more effective than the use of these drugs alone.

Anwer and Alghadir [29] applied a program including isometric quadriceps, straight leg raising, and isometric hip adduction exercise 5 days a week for a period of 5 weeks, whereas the control group did not perform any exercise program with 42 patients having knee OA. Numerical rating scale (NRS), a strength gauge, and reduced WOMAC index were measured. In between-group comparisons, the maximum isometric quadriceps strength, reduction in pain intensity, and improvement in function in the isometric exercise group at the end of the 5th week were significantly greater than those of the control group (*p* < 0.05).

Huang et al. [52] randomized patients with OA as an exercise treatment group (128 patients) and a traditional treatment control group (122 patients). Quadriceps isometric contraction exercise was used in the test group, and local physiotherapy and oral nonsteroidal anti-inflammatory drugs were used in the control group. The cases were evaluated with VAS and WOMAC before treatment, and after 1 and 3 months in posttreatment, it was reported that joint pain was effectively relieved and knee joint function was improved with systematic quadriceps isometric contraction exercise.

A recent systematic review reported that current evidence displays that exercise therapy is superior to placebo in the short term for pain and function in OA [53].

As a result, it is possible to obtain positive results with quadriceps isometric exercises performed in different positions to reduce pain intensity and joint stiffness and increase physical function in patients with knee OA. In our study, significant improvements were obtained in both groups in pain, stiffness, and physical function scores. Quadriceps isometric exercises performed only in extension were found to be more beneficial in reducing joint stiffness than those performed in flexion.

Because a combined physical therapy program (TENS, HP, US) was applied to all patients along with isometric exercises, it cannot be concluded that quadriceps isometric exercises have positive effects only on the treated knees. Combined physical therapy applications in knee OA improve pain and physical function. In this treatment, it was determined that adding isometric exercise to the quadriceps muscle was beneficial, especially regarding pain. Isometric exercise applied to the quadriceps muscle can be recommended in patients with knee OA who have difficulty to exercise and are contraindicated due to their systemic condition. Thus, the symptoms of the patients can be reduced and their physical function can be increased. In order to increase the success of treatment in knee OA, there is a need for new studies involving larger patient groups in which risk factors are evaluated.

The study was limited due to the implementation of strengthening exercises for only the extensor muscles of the patients, the lack of evaluation of the quadriceps muscle strength with the isokinetic system, and the low number of patients included. Besides, further studies are needed to see the long-term follow-up effects of the interventions for definitive results.

Quadriceps isometric exercises applied in two different positions in patients with knee OA reduce the severity of knee pain and joint stiffness and increase physical function. When the results of the quadriceps isometric exercises were compared, a statistically significant difference was found in the WOMAC-stiffness score in favor of group 1 studied in knee extension.

As a result, it is possible to obtain positive results in knee OA patients with the application of a combined physical therapy program in addition to the quadriceps isometric exercises to reduce pain and stiffness and increase physical function in patients with knee OA.

## Figures and Tables

**Table 1:** Demographic characteristics of patients.

	Group I (*n* = 14)	Group II (*n* = 15)	*p* value
Age (years)	59.2 ± 6.3	60.5 ± 7.2	0.511
Gender			
Male (*n*)	2 (%)	5 (%)	0.390
Female (*n*)	12 (%)	10 (%)	
BMI (kg/m^2^)	30.2 ± 2.7	31.5 ± 3.5	0.295
Duration of complaints (years)	3.6 ± 2.1	4.3 ± 2.1	0.390
Occupation			
Housewife	9	7	0.630
Retired	3	5	
Employee	2	3	
Education			
Illiterate	2	2	0.645
Literate	5	4	
Primary school	3	5	
Elementary school	3	1	
High school	1	2	
University	0	1	

Data are presented as mean ± standard deviation or *n* (%). BMI: body mass index. Group 1 (quadriceps isometric exercise was performed in knee extension), group 2 (quadriceps isometric exercise was performed in knee flexion).

**Table 2:** Baseline characteristics of groups.

	Group I (*n* = 14)	Group II (*n* = 15)	*p* value
VAS rest	3.1 ± 1.9	2.4 ± 2.5	0.204
VAS activity	7.7 ± 1.7	6.5 ± 2.5	0.157
WOMAC pain (0-20)	10.4 ± 2.4	9.4 ± 4.3	0.264
WOMAC stiffness (0-8)	3.2 ± 1.7	2.6 ± 2.3	0.320
WOMAC function (0-68)	33.8 ± 11.6	29.9 ± 11.4	0.370
WOMAC global (0-96)	47.4 ± 14.6	41.9 ± 16.4	0.394

Data are presented as mean ± standard deviation. VAS: visual analog scale; WOMAC: the Western Ontario and McMaster Universities Osteoarthritis Index. Group 1 (quadriceps isometric exercise was performed in knee extension), Group 2 (quadriceps isometric exercise was performed in knee flexion).

**Table 3:** Comparison of the VAS and WOMAC scores after the treatment.

	Group I (*n* = 14)				Group II (*n* = 15)				Between groups' *p* value
	Baseline	4^th^ week	Δ	*p* value	Baseline	4^th^ week	Δ	*p* value	
VAS rest	3.1 ± 1.9	1.3 ± 1.3	−1.8 ± 1.2	**0.001**	2.4 ± 2.5	1.0 ± 1.4	−1.4 ± 1.5	**0.003**	0.190
VAS activity	7.7 ± 1.7	3.9 ± 1.9	−3.8 ± 1.2	**0.001**	6.5 ± 2.5	3.1 ± 2.7	−3.3 ± 2.0	**0.001**	0.358
WOMAC pain (0-20)	10.4 ± 2.4	6.7 ± 2.4	−3.7 ± 1.7	**0.001**	9.4 ± 4.3	5.9 ± 3.9	−3.5 ± 1.5	**0.001**	0.516
WOMAC stiffness (0-8)	3.2 ± 1.7	1.6 ± 1.1	−1.6 ± 0.8	**0.001**	2.6 ± 2.3	1.7 ± 1.9	−0.9 ± 0.8	**0.002**	**0.035**
WOMAC function (0-68)	33.8 ± 11.6	20.5 ± 6.8	−13.3 ± 6.7	**0.001**	29.9 ± 11.4	18.3 ± 9.5	−11.6 ± 5.4	**0.001**	0.615
WOMAC global (0-96)	47.4 ± 14.6	28.8 ± 9.2	−18.6 ± 7.9	**0.001**	41.9 ± 16.4	26.5 ± 13.7	−15.3 ± 6.3	**0.001**	0.370

Data are reported as mean ± standard deviation. VAS: visual analog scale, WOMAC: the Western Ontario and McMaster Universities Osteoarthritis Index. Group 1 (quadriceps isometric exercise was performed in knee extension), Group 2 (quadriceps isometric exercise was performed in knee flexion).

## Data Availability

The datasets generated during and/or analysed during the current study are available from the corresponding author on reasonable request.
